# Prefecture-specific prevalence of overweight/obesity is associated with regional variation in the incidence of treated ESKD in Japan

**DOI:** 10.1007/s10157-022-02284-z

**Published:** 2022-10-08

**Authors:** Minako Wakasugi, Ichiei Narita

**Affiliations:** 1 Department of Inter-Organ Communication Research, 1-757 Asahimachi, Chuo-ku, Niigata, 951-8510 Japan; 2 Division of Clinical Nephrology and Rheumatology, Niigata University Graduate School of Medical and Dental Sciences, Niigata, Japan

**Keywords:** Dialysis, General population, Obesity, Regional variation, Standardized incidence ratio

## Abstract

**Background:**

Overweight/obesity is a significant risk factor for chronic kidney disease and end-stage kidney disease (ESKD) in the general population. This study evaluated the impact of sex- and prefecture-specific prevalence of overweight/obesity on standardized incidence rates (SIRs) of treated ESKD in Japan.

**Methods:**

We conducted an ecological study of all prefectures in Japan (*n* = 47) using data from the Japanese Society of Dialysis Therapy, national census, the NDB Open Data, and the Statistics of Physicians, Dentists and Pharmacists. We calculated the prevalence of overweight/obesity and proteinuria, standardized mortality ratio, and ratio of nephrology specialists for each prefecture, and explored associations of these variables with sex- and prefecture-specific SIRs of treated ESKD using bivariate association analysis, multiple regression analysis, and structural equation modeling (SEM).

**Results:**

Prefecture-specific SIRs ranged from 0.72 to 1.24 for men and 0.69–1.41 for women. Prefecture-specific SIRs were significantly correlated with both the prevalence of overweight/obesity and prevalence of proteinuria. The prevalence of overweight/obesity showed direct, positive, and significant associations with prefecture-specific SIRs in men (standardized estimate (*β*) = 0.43, *p* < 0.001) and women (*β* = 0.40, *p* < 0.001). The prevalence of proteinuria showed a significant association with prefecture-specific SIRs only in women (*β* = 0.33, *p* = 0.01). The SEM models explained 26% of the variance in SIR for men and 28% for women.

**Conclusions:**

Our findings provide evidence that the prefecture-specific prevalence of overweight/obesity in Japan can explain regional variation in prefecture-specific SIRs of treated ESKD in both sexes.

## Introduction

The worldwide prevalence of overweight/obesity has been rising [1]. High body mass index (BMI) is a significant risk factor for chronic kidney disease (CKD) and end-stage kidney disease (ESKD) in the general population [2–5]. A large, individual participant-level meta-analysis of observational studies by the Chronic Kidney Disease Prognosis Consortium (CKD-PC) showed that higher BMI (> 25 kg/m^2^) is associated with an increased risk of glomerular filtration rate (GFR) decline in general population cohorts [2]. A large retrospective cohort study also reported a strong graded relationship between the risk of ESKD and elevated BMI starting at a BMI of 25.0 kg/m^2^ [3]. Moreover, increased BMI is associated with an increased incidence of acute kidney injury requiring renal replacement therapy [6]. Furthermore, proteinuria, a strong risk factor for ESKD in the general population [7], is a consequence of overweight/obesity [5]. These findings collectively suggest that areas with a high prevalence of overweight/obesity may have an increased incidence of ESKD.

Japan, a country with a high incidence of treated ESKD [8], has substantial regional variation in the incidence of treated ESKD [9–14] as well as adult mean BMI [15], despite a uniform health care and insurance system [16] and low ethnic and racial diversity. The driving force behind the high incidence of treated ESKD in some areas may be the high prevalence of overweight/obesity. The two leading causes of dialysis in Japan are diabetes and hypertension [17], both of which are consequences of overweight/obesity. To date, however, no study has examined the association between the prevalence of overweight/obesity and incidence of treated ESKD at the prefecture level.

This ecological study aimed to examine the relationship between the prefecture-specific prevalence of overweight/obesity and proteinuria and standardized incidence rates (SIRs) of treated ESKD in Japan. We also evaluated the ratio of nephrology specialists to all physicians in each prefecture as a surrogate indicator for the quality of CKD care, as well as standardized mortality rates (SMRs) of the general population as a competing risk. We tested the hypothesis that regional variation in the incidence of treated ESKD among the Japanese general population would be explained, in part, by prefecture-specific prevalence of overweight/obesity.

## Materials and methods

### Study design and data source

This ecological cross-sectional study used data from four sources of nationwide open data, namely, the annual survey of the Japanese Society for Dialysis Therapy Renal Data Registry (JRDR), national vital statistics, the National Database of Health Insurance Claims and Specific Health Checkups of Japan (NDB) Open Data, and Statistics of Physicians, Dentists and Pharmacists, all of which except for the NDB Open Data are complete surveys.

An incident case of ESKD was defined as a patient with loss of kidney function that resulted in maintenance dialysis therapy including both hemodialysis and peritoneal dialysis [18]. Because the number of pre-emptive kidney transplant patients is small in Japan [19], this definition covers almost all ESKD patients for whom renal replacement therapy is initiated in Japan. In 2017, the number of pre-emptive kidney transplants in Japan was 662 [19], whereas the number of incident dialysis patients was 40,959 [17]. Incident cases of ESKD were extracted from annual data of the JRDR for 2016 and 2017 [17, 20] using the Web-based Analysis of Dialysis Data Archives (WADDA) system. Details of registry data collection techniques and characteristics of this dialysis population have been described elsewhere [17, 20]. In brief, the JRDR collects data every year through questionnaire surveys sent to all dialysis facilities in Japan.

The population and number of deaths for each prefecture during the same 2-year period were obtained from the national vital statistics provided by the Ministry of Health, Labour and Welfare.

Numbers of people with proteinuria and overweight/obesity in each prefecture for 2016 and 2017 were obtained from the NDB Open Data [21]. Details of the NDB Open Data have been described elsewhere [22]. In brief, the NDB, a large national administrative claims database, refers to the National Database of Health Insurance Claims and Specific Health Checkups of Japan operated by the Ministry of Health, Labour and Welfare. The NDB Open Data were constructed by aggregating a part of the NDB without any confidential information [22]; therefore, researchers using the NDB Open Data cannot access patient- or facility-level information. We used data from individuals who participated in the Specific Health Checkup program, which is available for insured persons and their dependents aged 40–74 years in Japan.

Numbers of nephrology specialists and all physicians in each prefecture were extracted from the Statistics of Physicians, Dentists and Pharmacists reported by the Ministry of Health, Labour and Welfare. We used data from 2016 since the statistics are reported biannually.

The present study was conducted according to the principles of the Declaration of Helsinki. All data used in the analyses were anonymized and included no individual patient data. Thus, ethical review and informed consent were not required.

### Statistical analysis

We analyzed data separately for men and women, as sex differences exist in the incidence of ESKD in Japan [23, 24]. Sex-specific SIR was calculated as the ratio of the observed number of treated ESKD patients to the expected number of incident ESKD cases using the indirect method previously described [25]. We also calculated sex-specific SMR as the ratio of the observed number of deaths to the expected number of deaths in the general population using the indirect method previously described [26–29].

Sex- and prefecture-specific prevalence of proteinuria was calculated as the proportion of health checkup participants with proteinuria divided by the total number of the health checkup participants by sex and prefecture. Proteinuria was defined as a dipstick urinalysis score of 1 + or greater (equivalent to ≥ 30 mg/dL). Sex- and prefecture-specific prevalence of overweight/obesity was calculated as the proportion of health checkup participants with overweight/obesity divided by the total number of the health checkup participants by sex and prefecture. Overweight/obesity was defined as BMI ≥ 25 kg/m^2^, the standard World Health Organization cut-off [30]. We used the average percentage for the 2 years (2016 and 2017). Prefecture-specific ratio of nephrology specialists to all physicians was calculated as the number of nephrology specialists divided by the total number of all physicians by prefecture.

Pearson’s correlation coefficients were used to evaluate the relationship between variables. We considered a coefficient < 0.1 as a negligible correlation, 0.1–0.39 as weak, 0.4–0.69 as moderate, 0.7–0.89 as strong, and 0.9–1 as very strong [31].

Hierarchical multiple regression was adopted to clarify further the comparative effects of sex- and prefecture-specific prevalence of overweight/obesity and proteinuria and prefecture-specific ratio of nephrology specialists on sex- and prefecture-specific SIRs. In Model 1, sex- and prefecture-specific prevalence of proteinuria was first put into the regression model as an independent variable. In Model 2, sex- and prefecture-specific prevalence of overweight/obesity was added. In Model 3, prefecture-specific ratio of nephrology specialists was added to the model. Adjusted R-squared values were used to determine the percentage of variation explained by the independent variable(s) that affects the dependent variables. Multicollinearity of independent variables was assessed by variation inflation factor (VIF). A VIF of 10 is considered excessive, while a VIF as low as 4 has been used to indicate high levels of multicollinearity between predictor variables.

Structural equation modeling (SEM) was performed to evaluate the impact of the association between the prevalence of overweight/obesity, prevalence of proteinuria, and ratio of nephrology specialists on sex- and prefecture-specific SIRs. The model for SIRs included variables based on the results of the correlation analysis.

All reported *P* values were two-sided, and values < 0.05 were considered statistically significant. Statistical analyses were performed using AMOS version 27 (IBM Inc, Armonok, NY) and IBM SPSS Statistics version 27 (SPSS, Chicago).

## Results

During the 2-year study period, 52,183 men (0.42 per 1000 person-years) and 23,853 women (0.18 per 1000 person-years) started dialysis for ESKD in Japan, and 1,365,503 men (11.1 per 1000 person-years) and 1,282,812 women (9.9 per 1000 person-years) died. In 2016, 304,759 medical doctors worked at medical facilities in Japan, and of these, 6,850 (2.2%) were nephrology specialists. Urinalysis data were available for 30,561,231 men and 25,400,390 women, accounting for 51.4% and 41.8% of the same-sex population in the age range 40–74 years in Japan, respectively; of these, 1,516,375 men (5.0%) and 667,572 women (2.6%) had proteinuria, respectively. BMI data were available for 30,659,889 men and 25,611,789 women, accounting for 51.6% and 42.1% of the same-sex population in the age range 40–74 years in Japan, respectively; of these, 10,346,302 men (33.7%) and 5,037,906 women (19.7%) were overweight/obese, respectively.

**Fig. 1:**
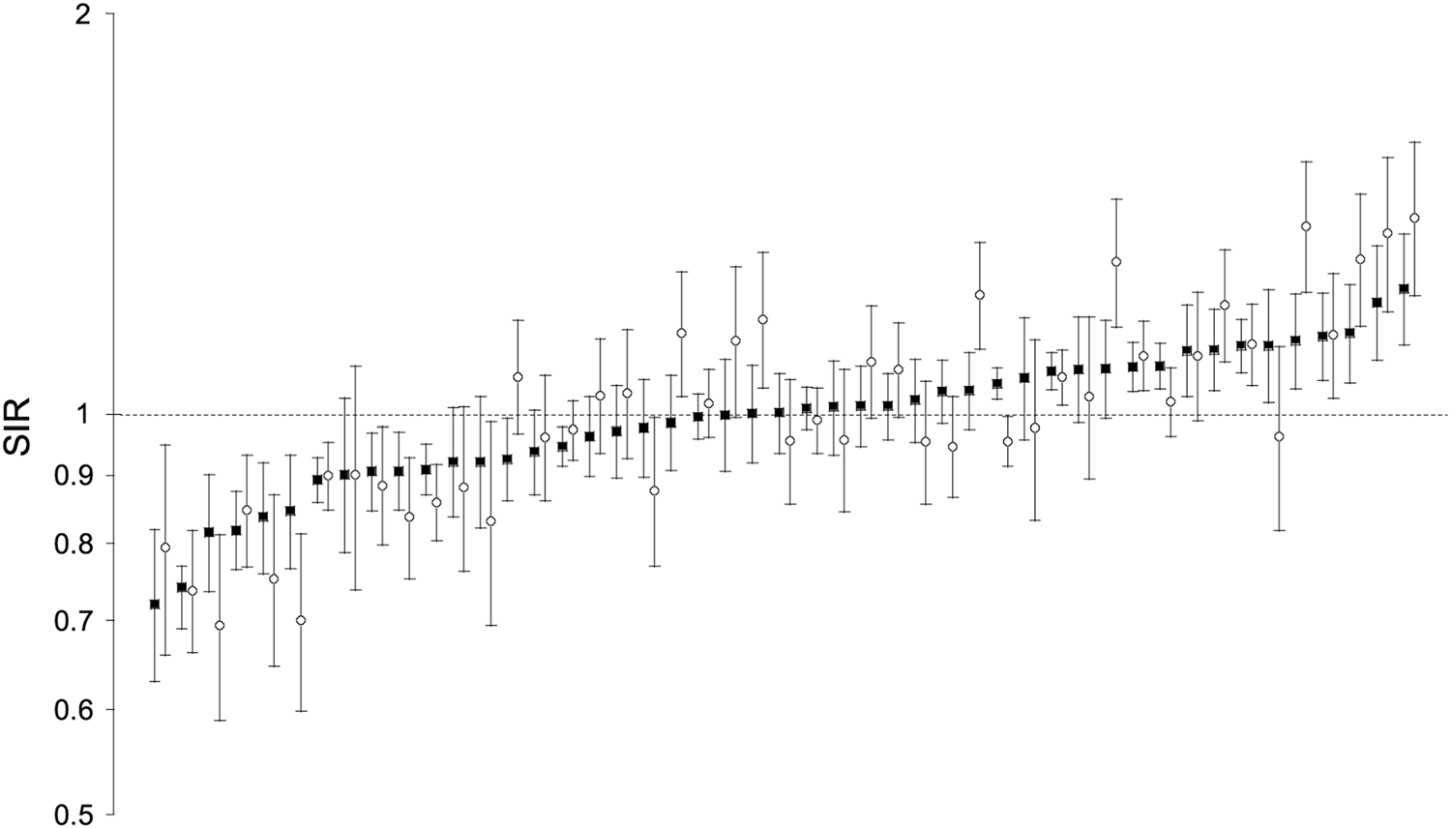
Age-adjusted standardized incidence rate ratios (SIRs) of treated ESKD among men (filled squares) and women (open circles) in the 47 prefectures. Error bars indicate the 95% confidence interval. Sex- and prefecture-specific SIRs among men are presented in ascending order. Prefecture names are not shown. *ESKD* end-stage kidney disease

**Fig. 2:**
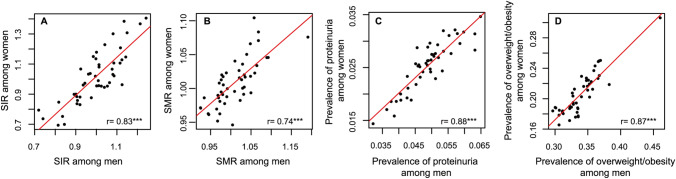
Scatter plots with correlation coefficients showing high correlations between men and women in **A** SIRs, **B** SMRs, **C** prevalence of proteinuria, and **D** prevalence of overweight/obesity in the 47 prefectures. *N* for all variables = 47. *Denotes significant Pearson’s correlation coefficients with ***P < 0.001. *SIR* standardized incidence ratio, *SMR* standardized mortality ratio

Descriptive characteristics of the study variables are shown in Table 1. Sex- and prefecture-specific SIRs ranged from 0.72 (95% CI, 0.63–0.82) to 1.24 (1.13–1.37) for men and 0.69 (0.59–0.81) to 1.41 (1.23–1.60) for women. In each prefecture, SIRs for men (filled squares) were close in value to SIRs for women (open circles), with a few exceptions (Fig. 1). Prefecture-specific SIRs for men were strongly correlated with those for women (*r* = 0.83, *p* < 0.001) (Fig. 2A). Prefecture-specific SMRs (Fig. 2B), prevalence of proteinuria (Fig. 2C), and prevalence of overweight/obesity (Fig. 2D) for men and women were also strongly correlated.

**Table 1:** Descriptive statistics for all variables used in this study

Variables	Mean (SD)	Min	Max
SIR of treated ESKD among men	1.00 (0.12)	0.72	1.24
SIR of treated ESKD among women	1.02 (0.18)	0.69	1.41
SMR of the general male population	1.01(0.05)	0.93	1.19
SMR of the general female population	1.01 (0.04)	0.95	1.10
Prevalence of proteinuria among men (%)	5.0 (0.7)	3.3	6.7
Prevalence of proteinuria among women (%)	3.8 (0.6)	2.3	5.1
Prevalence of overweight/obesity among men (%)	33.9 (2.8)	30.0	46.0
Prevalence of overweight/obesity among women (%)	20.6 (2.7)	16.6	30.6
Ratio of nephrology specialists (%)	1.2 (0.3)	0.6	1.9

*N* for all variables = 47

*ESKD* end-stage kidney disease, *SD* standard deviation, *SIR* standardized incidence ratio, *SMR* standardized mortality ratio

**Fig. 3:**
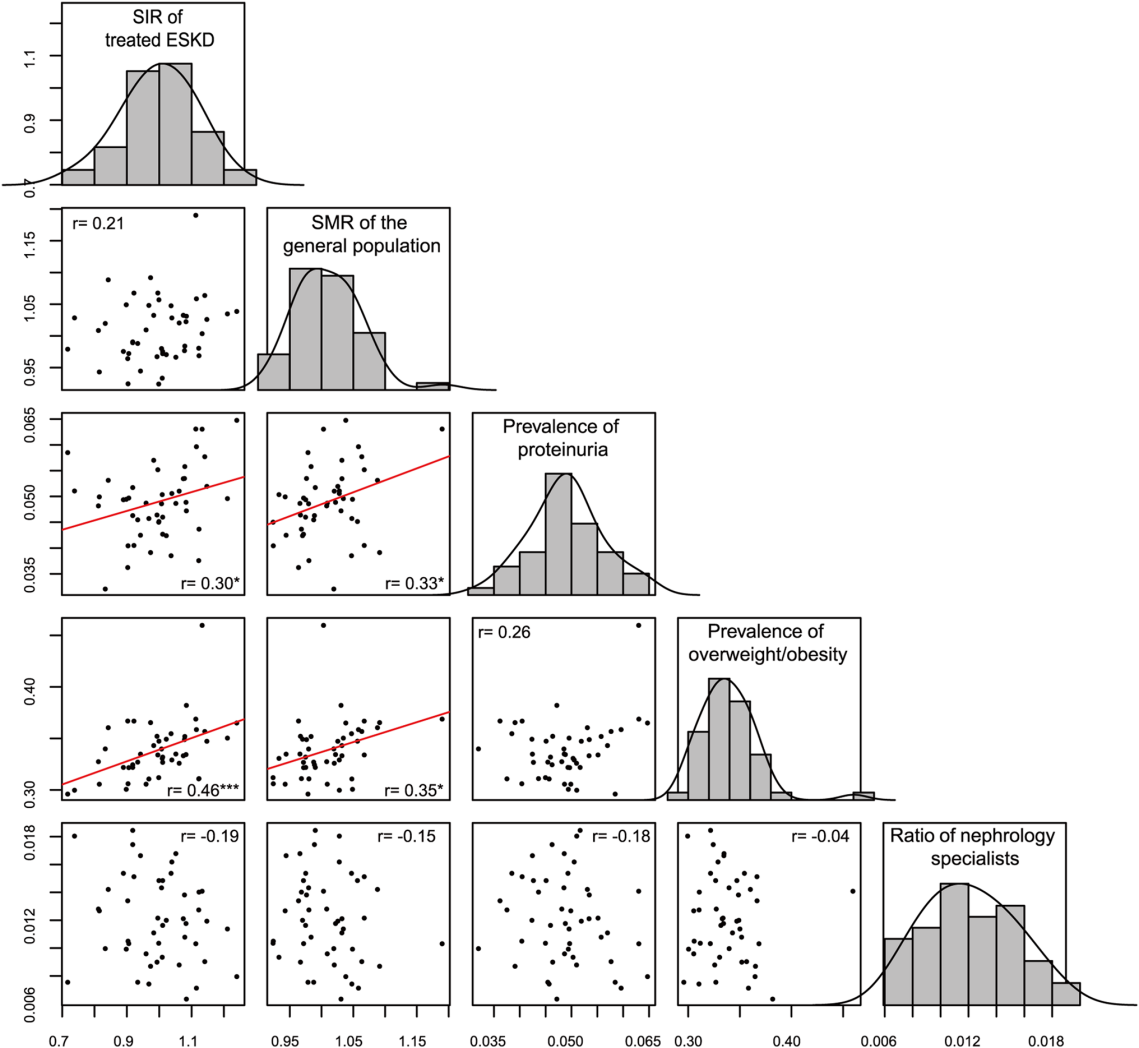
Scatter plots and histograms between SIRs, SMRs, prevalence of proteinuria, prevalence of overweight/obesity, and ratio of nephrology specialists among men in the 47 prefectures. *N* for all variables = 47. *Denotes significant Pearson’s correlation coefficients with *P < 0.05; **P < 0.01; ***P < 0.001. *ESKD* end-stage kidney disease, *SIR* standardized incidence ratio, *SMR* standardized mortality ratio

**Fig. 4:**
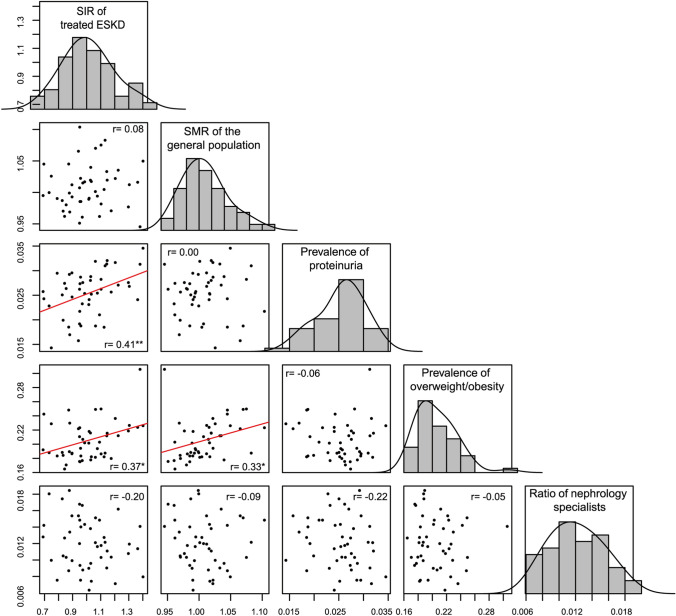
Scatter plots and histograms between SIRs, SMRs, prevalence of proteinuria, prevalence of overweight/obesity, and ratio of nephrology specialists among women in the 47 prefectures. *N* for all variables = 47. *Denotes significant Pearson’s correlation coefficients with *P < 0.05; **P < 0.01; ***P < 0.001. *ESKD* end-stage kidney disease, *SIR* standardized incidence ratio, *SMR* standardized mortality ratio

As shown in Fig. 3, prefecture-specific SIRs were weakly correlated with the prevalence of proteinuria (*r* = 0.30, *p* = 0.04), and moderately correlated with the prevalence of overweight/obesity (*r* = 0.46, *p* < 0.001), in men. Both the prevalence of proteinuria (*r* = 0.33, *p* = 0.02) and prevalence of overweight/obesity (*r* = 0.35, *p* = 0.02) were also weakly correlated with prefecture-specific SMRs in men. Similarly, in women, prefecture-specific SIRs were moderately correlated with the prevalence of proteinuria (*r* = 0.41, *p* = 0.004) and weakly correlated with the prevalence of overweight/obesity (*r* = 0.37, *p* = 0.01) (Fig. 4). In women, prefecture-specific SMRs were weakly correlated with the prevalence of overweight/obesity (*r* = 0.33, *p* = 0.03) but not that of proteinuria (*r* = 0.005, *p* = 0.97). No significant correlations were observed between prefecture-specific SIRs and SMRs in both sexes. Ratios of nephrology specialists were not correlated with prefecture-specific SIRs in both sexes.

Hierarchical linear regression analysis revealed that the prevalence of proteinuria was significantly associated with prefecture-specific SIRs in men (Table 2). Further adjustment for the prevalence of overweight/obesity, however, attenuated the association, and the prevalence of proteinuria was no longer significantly associated with prefecture-specific SIRs in men. Further adjustment for the ratio of nephrology specialists did not change the association between the prevalence of overweight/obesity and prefecture-specific SIRs in men.

**Table 2:** Summary of hierarchical regression analysis for variables predicting SIRs for men

Variables	Model 1		Model 2		Model 3	
	*β*	*P*	*β*	*P*	*β*	*P*
Prevalence of proteinuria among men	0.30	0.04	0.19	0.18	0.16	0.25
Prevalence of overweight/obesity among men			0.42	0.004	0.42	0.004
Ratio of nephrology specialists					− 0.15	0.28
Adjusted R^2^	0.07		0.21		0.22	
F for change in R^2^	4.30	0.04	9.44	0.004	1.19	0.28

*N* for all variables = 47

*β*: standardized regression coefficient, *SIR* standardized incidence ratio

The prevalence of proteinuria was significantly associated with prefecture-specific SIRs in women (Table 3). Further adjustment for the prevalence of overweight/obesity (Model 2) did not change the association, and both the prevalence of proteinuria and prevalence of overweight/obesity were significantly associated with prefecture-specific SIRs in women. Further adjustment for the ratio of nephrology specialists (Model 3) did not change these associations. Multicollinearity was assessed, and no VIF above 2 was observed.

**Table 3:** Summary of hierarchical regression analysis for variables predicting SIRs for women

Variables	Model 1		Model 2		Model 3	
	*β*	*P*	*β*	*P*	*β*	*P*
Prevalence of proteinuria among women	0.41	0.004	0.43	0.001	0.41	0.003
Prevalence of overweight/obesity among women			0.39	0.003	0.39	0.004
Ratio of nephrology specialists					− 0.09	0.48
Adjusted R^2^	0.15		0.29		0.28	
F for change in R^2^	9.07	0.004	9.86	0.003	0.51	0.48

*N* for all variables = 47

*β*: standardized regression coefficient, *SIR* standardized incidence ratio

**Fig. 5:**
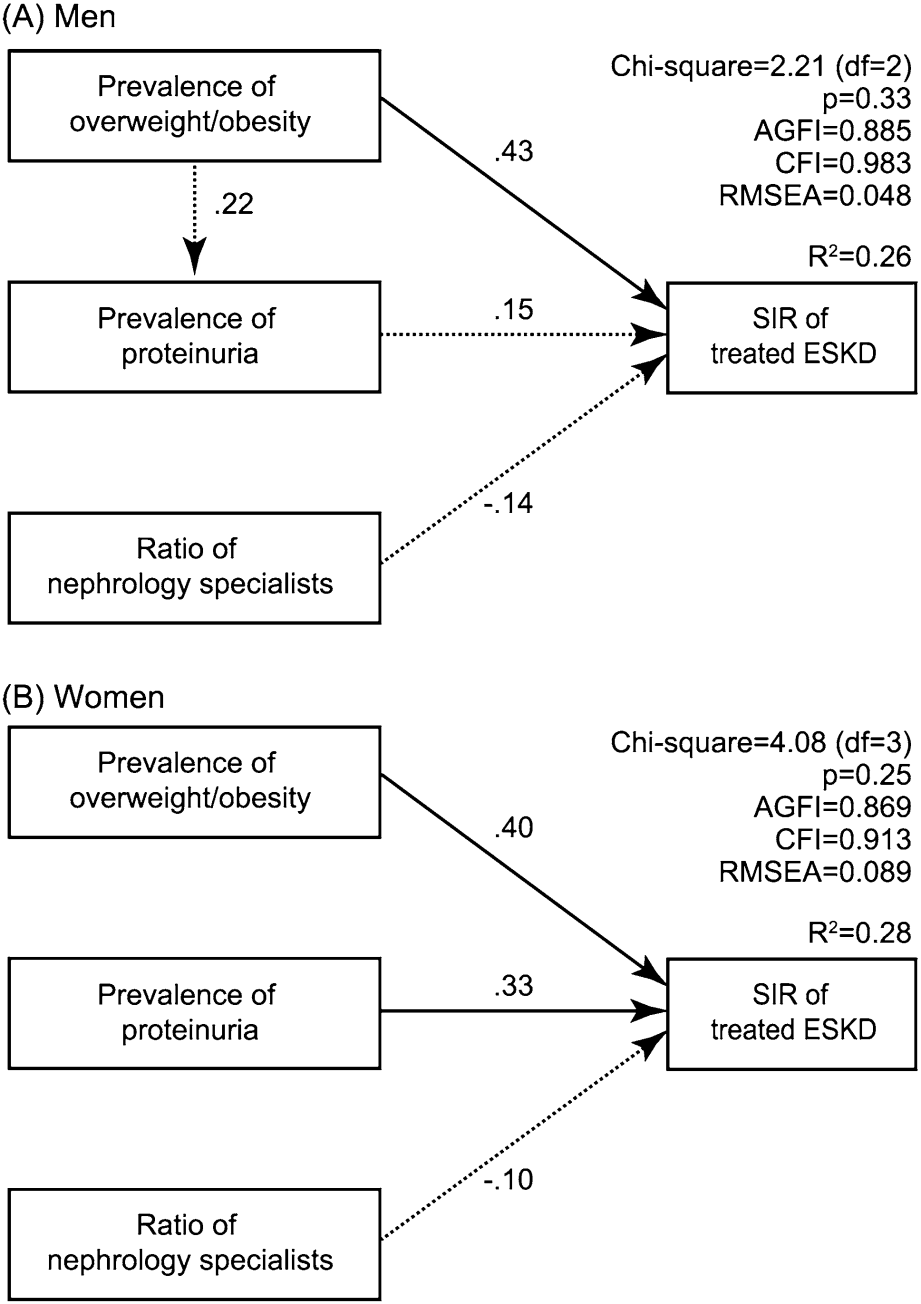
SEM models and standardized estimates. **A** Men; **B** Women. Solid lines indicate significant paths. Dashed lines indicate non-significant paths. The standardized regression coefficient is provided for each relationship. For simplicity, residual variance associated with independent variables is not represented. *ESKD* end-stage kidney disease, *SEM* structural equation modeling, *SIR* standardized incidence ratio

Based on the results above, hypothesis models for men (Fig. 5A) and women (Fig. 5B) were constructed. The fit of the male model to the data was adequate, with chi-square value = 2.21, df = 2, *p* = 0.33, CFI = 0.98, AGFI = 0.89, and RMSEA = 0.05, and this model explained 26% of the variance in prefecture-specific SIR for men. The path from the prevalence of overweight/obesity to prefecture-specific SIRs for men was positive (*β* = 0.43, *p* < 0.001), indicating that a higher prefecture-specific prevalence of overweight/obesity was significantly associated with a higher prefecture-specific SIR in men. For women, the fit of the model to the sample data was as follows: chi-square value = 4.08, df = 3, *p* = 0.25, CFI = 0.91, AGFI = 0.87, and RMSEA = 0.09. Both the prevalence of overweight/obesity (*β* = 0.40, *p* < 0.001) and prevalence of proteinuria (*β* = 0.33, *p* = 0.01) showed direct, positive, and significant associations with prefecture-specific SIRs in women. This model explained 28% of the variance in prefecture-specific SIR for women.

## Discussion

This ecological study with prefectures as the unit of analysis demonstrated correlations among prefecture-specific SIRs of treated ESKD, prevalence of overweight/obesity, and prevalence of proteinuria in men and women. Neither SMRs of the general population nor the ratio of nephrology specialists were correlated with prefecture-specific SIRs in both sexes. The prefecture-specific prevalence of overweight/obesity showed direct, positive, and significant associations with prefecture-specific SIRs in both sexes, while the prefecture-specific prevalence of proteinuria showed a direct, positive, and significant association with prefecture-specific SIRs only in women. Results of SEM showed that models consisting of the prevalence of overweight/obesity, prevalence of proteinuria, and ratio of nephrology specialists explain 26% of the variance in SIR for men and 28% for women.

Our data showed that the prefecture-specific prevalence of overweight/obesity was significantly and positively associated with prefecture-specific SIRs in both sexes, suggesting that preventing overweight/obesity has a significant impact on reducing the incidence of treated ESKD, irrespective of sex. This is not surprising because numerous population-based studies have shown an association between high BMI and both the development and progression of CKD [5]. Furthermore, overweight/obesity is an increasingly becoming common cause of CKD, even in the absence of metabolic abnormality [32]. Taken together, areas with a high incidence of treated ESKD may benefit from measures to reduce the prevalence of overweight/obesity.

To the best of our knowledge, this is the first study to report associations of the sex- and prefecture-specific prevalence of overweight/obesity and proteinuria with sex- and prefecture-specific SIRs of treated ESKD. Previous ecological studies have focused on regional variation in CKD practice patterns, such as the amount of money spent on angiotensin-converting enzyme (ACE) inhibitors [10], statins [11], and integrated therapies including erythropoietin [13]. Our results showed a non-significant path from the ratio of nephrology specialists as a surrogate indicator for the quality of CKD care to prefecture-specific SIRs in both sexes, suggesting that the prevalence of overweight/obesity and proteinuria, rather than regional variation in CKD practice patterns, has a significant impact on prefecture-specific SIRs in Japan. Recent evidence-based medicine and clinical guidelines [33, 34] might have minimized regional variation in CKD practice patterns, thereby reducing the influence of CKD practice patterns on SIRs. The increasing proportion of diabetes as the cause of ESKD may increase the influence of overweight/obesity on SIRs. The leading cause of ESKD in Japan has moved from glomerulonephritis to diabetes since 1998 [20].

In the present study, SEM revealed a significant path from the prefecture-level prevalence of proteinuria to prefecture-specific SIRs only in women. Proteinuria may occur due to glomerulonephritis in some female patients, and in fact, the percentage of chronic glomerulonephritis as the cause of ESKD was higher in women (18.0%) than in men (15.5%); contrarily, the percentage of diabetes as the cause of ESKD was lower in women (35.8%) than in men (45.6%) [20]. Thus, screening for proteinuria may offer an effective strategy to tackle the burden of CKD and ESKD, especially among women. This approach is also cost-effective [35].

This study has several strengths. First, since data were extracted from four nationwide sources, our findings are broadly generalizable to the Japanese population. Second, to the best of our knowledge, this is the first study to report associations of sex- and prefecture-specific prevalence of overweight/obesity and proteinuria with sex- and prefecture-specific SIRs of treated ESKD. Thus, our findings provide evidence that overweight/obesity is associated with the incidence of treated ESKD from a population-level perspective.

There are also several limitations worth noting. First, selection bias may exist because the NDB Open Data covered about half of the 40–74-year-old population in Japan. Furthermore, we did not consider the prefecture-specific participation rate for Specific Health Checkups. Thus, it is possible that the prefecture-specific prevalence of overweight/obesity or proteinuria in this study may not be the same as those of the entire population. The prefecture-specific prevalence in the 40–74-year-old population, however, can be calculated based on data from Specific Health Checkups, which are conducted every year. Evaluating and reducing the prefecture-specific prevalence of overweight/obesity or proteinuria in the 40–74-year-old population may bring benefits to areas with a high incidence of treated ESKD. Second, only patients who had undergone dialysis treatment were included, as data were not available for patients who had not initiated dialysis or had undergone pre-emptive kidney transplantation in Japan. The number of pre-emptive kidney transplants in Japan, however, is small, with only 662 patients being reported in 2017 [19]. Third, due to the limited sample size (*n* = 47), we could only use a limited number of parameters to estimate SEM models. Fourth, sex- and prefecture-specific SIRs and SMRs were adjusted only for age because individual patient data were lacking. Finally, since this was an ecological study, there is a risk of ecological fallacy, i.e., findings observed at the prefecture level may not be seen at the facility or individual level. However, numerous population-based studies have shown an association between high BMI and both the development and progression of CKD [5]. Thus, maintaining a healthy weight is undoubtedly important to prevent CKD as well as ESKD in the general population.

## Conclusions

This ecological study showed that a higher prefecture-specific prevalence of overweight/obesity is associated with a higher prefecture-specific SIR of treated ESKD in both sexes, and that a higher prefecture-specific prevalence of proteinuria is associated with a higher prefecture-specific SIR of treated ESKD only in women. Our results send a clear message to the general population and health professionals that maintaining a healthy weight is important to protect the kidneys.
